# Rapid characterization of feline leukemia virus infective stages by a novel nested recombinase polymerase amplification (RPA) and reverse transcriptase-RPA

**DOI:** 10.1038/s41598-021-01585-9

**Published:** 2021-11-11

**Authors:** Sitthichok Lacharoje, Somporn Techangamsuwan, Nuntaree Chaichanawongsaroj

**Affiliations:** 1 Program of Molecular Sciences in Medical Microbiology and Immunology, Department of Transfusion Medicine and Clinical Microbiology, Faculty of Allied Health Sciences, Chulalongkorn University, Bangkok, 10330 Thailand; 2 Department of Pathology, Faculty of Veterinary Science, Chulalongkorn University, Bangkok, 10330 Thailand; 3 Animal Virome and Diagnostic Development Research Group, Faculty of Veterinary Science, Chulalongkorn University, Bangkok, 10330 Thailand; 4 Research Unit of Innovative Diagnosis of Antimicrobial Resistance, Department of Transfusion Medicine and Clinical Microbiology, Faculty of Allied Health Sciences, Chulalongkorn University, 154 Rama I Road, Wangmai, Pathumwan, Bangkok, 10330 Thailand

**Keywords:** Microbiology, Infectious-disease diagnostics

## Abstract

Feline leukemia virus (FeLV) is a major viral disease in cats, causing leukemia and lymphoma. The molecular detection of FeLV RNA and the DNA provirus are important for staging of the disease. However, the rapid immunochromatographic assay commonly used for antigen detection can only detect viremia at the progressive stage. In this study, nested recombinase polymerase amplification (nRPA) was developed for exogenous FeLV DNA provirus detection, and reverse transcriptase polymerase amplification (RT-RPA) was developed for the detection of FeLV RNA. The approaches were validated using 108 cats with clinicopathologic abnormalities due to FeLV infection, and from 14 healthy cats in a vaccination plan. The nRPA and RT-RPA assays could rapidly amplify the FeLV template, and produced high sensitivity and specificity. The FeLV detection rate in regression cats by nRPA was increased up to 45.8% compared to the rapid immunochromatographic assay. Hence, FeLV diagnosis using nRPA and RT-RPA are rapid and easily established in low resource settings, benefiting FeLV prognosis, prevention, and control of both horizontal and vertical transmission.

## Introduction

Feline leukemia virus (FeLV) is an enveloped positive single-stranded retrovirus which is widely distributed in domestic cats, and occurs occasionally in closely related wild felids^1^. The virus causes fatal diseases, especially cytopenia and hemopoietic tumors including acute myeloid leukemia, thymic lymphoma, multicentric lymphoma, myelodysplastic syndromes, myelodegenerative disease, and aplastic anemia^2,3^. Coinfection with FeLV was found in more than 90% of cats with feline infectious peritonitis and more than 3.5% of those with feline immunodeficiency virus (FIV), increasing the severity of the symptoms and the occurrence of complications^4^. The prevalence of FeLV infections varies among geographical areas, occurring in about 31.3% of cats in Malaysia^5^, 24.5% in Thailand^6^, 14.2% in Iran^7^, 3.6% in Germany^8^, and 3.4% in Canada^9^. Horizontal transmission occurs by direct contact with infected cats via oronasal exposure to saliva by licking, mutual grooming, sharing food dishes or water bowls, and biting, and also from exposure to feces, milk, and by blood transfusion^10,11^. Although vaccination reduces FeLV infection in unexposed cats, it does not provide 100% protection. Moreover, FeLV vaccination does not prevent DNA provirus integration after exposure. The best methods of prevention are feeding cats in a closed area, separating an infected individual in a multicat household or animal shelter, and screening blood for the virus before blood transfusion^12^.

The characteristics of FeLV infection have been classified into three stages based on provirus, plasma RNA, and antibody detection. The absence of FeLV proviral and RNA antigens is categorized as an abortive infection in either anti-FeLV antibody positive cats that have a low dose exposure and self-clearing of the virus by the immune system, or as antibody negative in unexposed cats. A regressive infection is categorized by undetectable antigenemia, and FeLV RNA converts to exogenous DNA provirus, which usually integrates into the chromosomes of the germ cells. The incorporated DNA provirus becomes endogenous DNA in the host chromosome, leading to vertical transmission. The disease may relapse in regressor cats under immunosuppressive conditions^13^. Progressive infections are categorized by detectable antigenemia, FeLV RNA, and DNA provirus, since the virus persistently sheds into the circulation and tissues. Such progressive stage infected cats are a major reservoir for horizontal transmission. About 50% of cats with persistent viremia die within 2 years, and 80% die within 3 years^14^. Although antiviral drugs and chemotherapy can retard mortality, they cannot cure the infection or prevent mortality. Consequently, early diagnosis is beneficial in stopping or reducing the cycle of horizontal and vertical transmission in feline populations, and in managing the clinical outcomes.

Routine diagnosis of FeLV infection is usually performed by detection of the p27 antigen using rapid immunochromatography. However, false negative results occur in early viremia and latent infections, as occurs in detection using ELISA, immunofluorescence, and virus isolation techniques, because these assays depend upon viremia or the presence of antibodies in the blood and other secretions^12^. False positive results have been demonstrated in three point of care FeLV p27 test kits, using either single or combination tests^15^. Methods based on the detection of FeLV DNA provirus or RNA are therefore more reliable, and are recommended as the gold standard approach. A number of molecular methods have been developed, including conventional PCR, nested (n)PCR^16–18^ and real-time quantitative PCR (rtq)PCR^19,20^. Although PCR-based methods are superior to serological assays and viral isolation, they require the use of a costly thermal cycler and trained personnel, and are time consuming.

Recombinase polymerase amplification (RPA), a novel isothermal nucleic acid amplification, requires a simple heating instrument or even body heat to generate the reaction^21^. The RPA process relies on three key proteins: recombinase, single-stranded DNA binding protein (SSB), and polymerase. The nucleoprotein, comprised of each primer and recombinase, binds to complementary sequences in the targets. The SSB binds to the dissociated DNA strand, stabilizes it, and prevents DNA reannealing. The polymerase exponentially amplifies the target sequence. The RPA reaction can be achieved in 20 min or less at a constant temperature of 37 °C–42 °C, and have high specificity and sensitivity^22^. The principle of primer design for nRPA is similar to that for nPCR. The two primers are designed for two consecutive RPA reactions. The first set of primers are designed to anneal to specific target sequences. The primary RPA products are used as the templates for secondary RPA reactions. The second sets of primers are design to anneal downstream from the first set of primers and amplify the internal target of initial amplicon^23^. The sensitivity and specificity of RPA amplification could be enhanced as in nPCR. Therefore RPA-based methods have become promising tools for the detection of various pathogens, particularly in laboratories that are not well equipped, or for field diagnosis. In this paper we described the development and validation of nRPA and reverse transcriptase (RT-RPA) assays for the detection of FeLV DNA provirus and RNA. Both FeLV RPA assays can be used in veterinary hospitals for rapid diagnosis, which is required in order to enable effective prevention and infection control.

## Results

### Development of the nRPA

**Figure 1:**
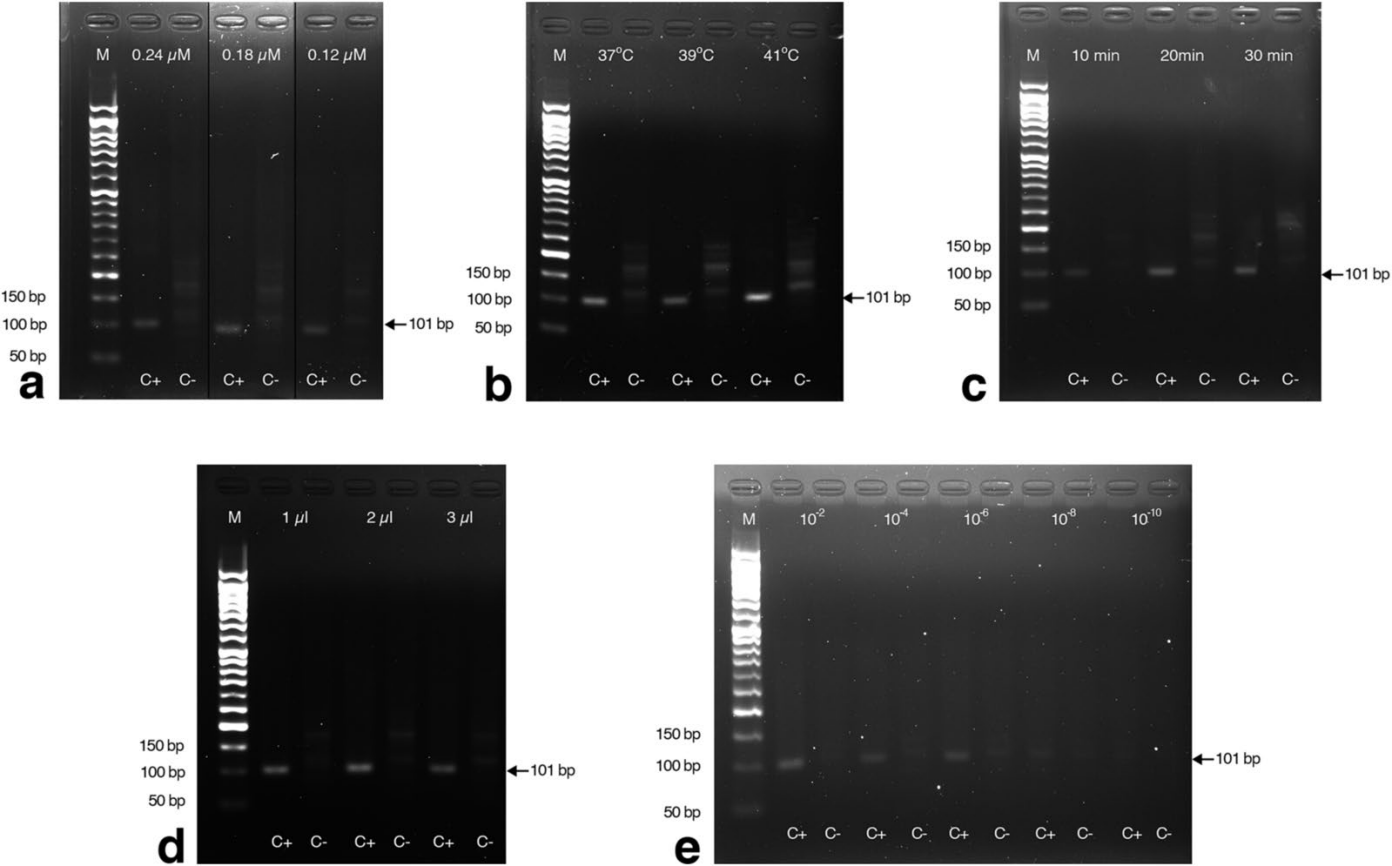
Optimization of FeLV DNA provirus detection by nRPA. Representative gel images showing the results from the nRPA reaction at different (**a**) inner primer concentrations (0.24, 0.18, and 0.12 µM) incubated at 37 °C for 20 min; (**b**) temperatures (37 °C, 39 °C, and 41 °C) incubated for 20 min with 0.36 µM inner primers; (**c**) incubation times (10, 20, and 30 min) at 37 °C with 0.18 µM inner primers; and (**d**,**e**) dilutions of primary RPA products at 37 °C for 10 min with 0.18 µM inner primers. C+, positive DNA control; C−, no-template control; M, molecular weight marker. Gel in (**a**) was cropped, see Supplementary Fig. S1 for the full-length picture.

The pGEM-T vector harboring the 145 bp of the U3LTR FeLV fragment was used as a template to optimize the nRPA assay. After completion of the first RPA reaction, the RPA product was diluted tenfold and subjected to a second RPA reaction under various conditions. The expected amplicon band of 101 bp was visible at all tested concentrations of the inner primer (0.24, 0.18, and 0.12 µM), temperature (37 °C, 39 °C, and 4 °C), incubation time (10, 20, and 30 min) and volume of primary template (1, 2, and 3 µL) (Fig. 1a–d). However, nonspecific bands appeared in the nontemplate control when the incubation time was longer than 10 min and when the template was present in high amounts. Consequently, the primary RPA product was serially diluted 100-fold from 10^−2^ to 10^−10^, and the expected amplicon band was observed at a 10^−2^ template dilution (Fig. 1e) without any nonspecific bands. Therefore, the optimal nRPA conditions was selected to be 37 °C for 10 min using 0.18 µM of inner primer concentration and 100-fold dilution of the primary RPA product.

### Development of RT-RPA

**Figure 2:**
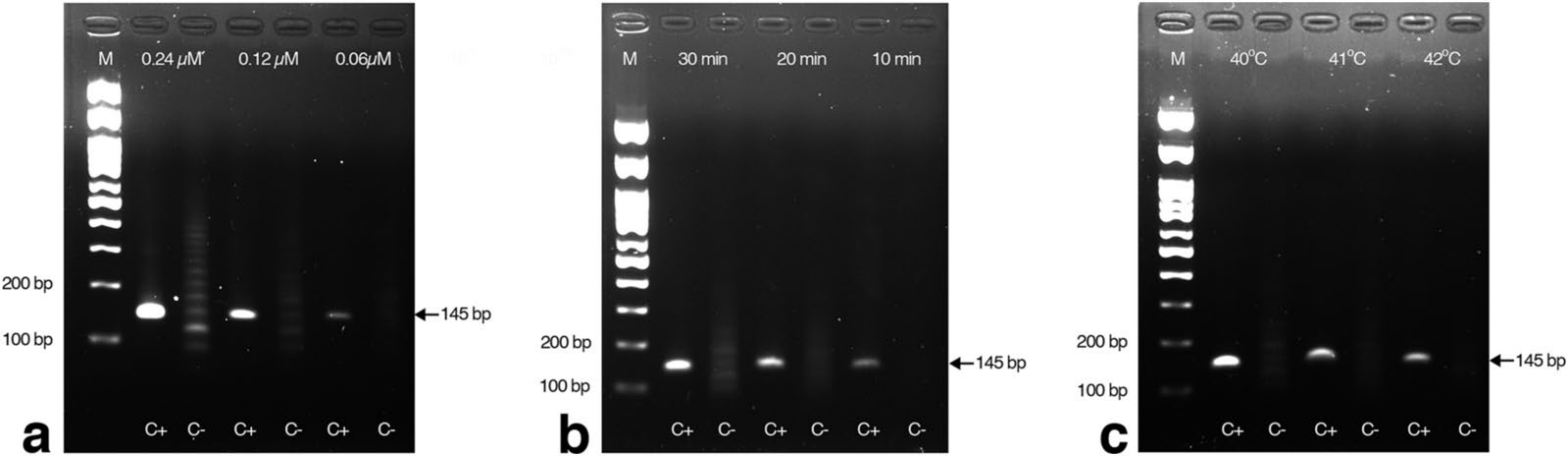
Optimization of FeLV RNA detection by RT-RPA. Representative gel images showing the results from the RT-RPA reactions at different (**a**) primer concentrations (0.24, 0.12, and 0.06 µM) incubated at 40 °C for 20 min; (**b**) incubation times (10, 20, and 30 min) at 40 °C with 0.12 µM primers; and (**c**) temperatures (40 °C, 41 °C, and 42 °C) for 10 min. C+, positive DNA control; C−, no template control; M, molecular weight marker.

FeLV RNA extracted from Nobivac^®^ Feline 2-FeLV vaccine was utilized for the RT-RPA optimization. The primers were the same as those used in the primary RPA and PCR reactions to amplify the 145 bp of exogenous U3LTR FeLV fragment. Although all primer concentrations tested (0.24, 0.12, and 0.06 µM), incubation times (10, 20, and 30 min), and temperatures (40 °C, 41 °C, and 42 °C) showed positive results (Fig. 2a–c), an incubation time of longer than 10 min caused the appearance of nonspecific bands in the no-template control. Hence, the appropriate conditions were selected as 40 °C, 10 min, using 0.12 µM primers, as this resulted in the most obvious positive amplicon band with no primer-dimer in the negative control.

### Validation of nRPA in clinical samples

From the 122 clinical samples tested, the FeLV provirus was detected by RPA and/or nRPA in all of the 90 samples found to be positive by PCR and/or nPCR. The positive RPA or PCR samples revealed a 145 bp amplicon, with the negative results being subsequently retested by nRPA or nPCR to yield the expected 101 bp amplicon in positive samples. There was an almost invisible positive band in seven negative nRPA samples with negative nPCR results and in four negative nPCR with negative nRPA results. These samples were retested using both techniques, and the negative results were confirmed. The results are summarized in Table 1. Detection of FeLV provirus by PCR revealed 73 positive and 49 negative results, and 17 of these 49 negative PCR were then found to be positive by nPCR, giving a total of 90/122 positive samples. Detection of FeLV provirus by RPA revealed 70 positive and 52 negative results, with 20 out of these 52 negative RPA samples being found to be positive by nRPA, giving a total of 90/122 positive samples. Thus, 90 samples were found to be positive by both the RPA/nRPA and the PCR/nPCR approaches, with nRPA and nPCR having a higher sensitivity than RPA and PCR.

**Table 1:** Testing of clinical samples by PCR, nPCR, RPA, and nested RPA.

Results	FeLV provirus			FeLV RNA		
	PCR	nPCR	PCR + nPCR	RPA	nRPA	RPA + nRPA
Positive (n)	73	17	90	70	20	90
Negative (n)	49	32	32	52	32	32
Total (n)	122	49	122	122	52	122

Comparisons of the results obtained from RPA with those from PCR, and of the results obtained from RPA plus nRPA with those from PCR plus nPCR for FeLV provirus detection are summarized in Tables 2 and 3, respectively. The RPA assay resulted in three false negative results compared to the PCR assay, which was taken to be the gold standard. The diagnostic performance of RPA compared to PCR produced 95.89% (95% confidence intervals [CI] of 88.46–99.14) sensitivity, 100% (95% CI of 92.89–100) specificity, and a kappa value of 0.949 (CI of 0.893–1.000). The nRPA demonstrated one false negative and one false positive result compared to nPCR. The diagnostic performance of RPA plus nRPA compared to PCR plus nPCR was 98.89% (95% CI of 93.96–99.97) sensitivity, 96.88% (95% CI of 83.78–99.92) specificity, and a kappa value of 0.958 (CI of 0.899–1.000). The accuracies of the RPA vs. PCR and RPA plus nRPA vs. PCR plus nPCR were 97.54% and 98.36%, respectively. The results supported the contention that the novel nRPA assays increased the sensitivity of FeLV provirus detection.

**Table 2:** Comparison of RPA and PCR for FeLV provirus detection in clinical samples.

PCR	RPA		Total	Sensitivity, % (95%CI)	Specificity, % (95%CI)	PPV, % (95%CI)	NPV, % (95%CI)	Kappa
	Positive	Negative						
Positive	70	3	73	95.89 (88.46–99.14)	100 (92.89–100)	100 (92.89–100)	94.23 (84.36–98.02)	0.949
Negative	0	49	49					
Total	70	52	122

**Table 3:** Comparison of RPA plus nRPA with PCR plus nPCR for FeLV provirus detection in clinical samples.

PCR + nPCR	RPA + nRPA		Total	Sensitivity, % (95%CI)	Specificity, % (95%CI)	PPV, % (95%CI)	NPV, % (95%CI)	kappa
	Positive	Negative						
Positive	89 (PCR/RPA 70) (nPCR/nRPA 16) (nRPA 3)	1 (nRPA)	90	98.89 (93.96–99.97)	96.88 (83.78–99.92)	98.89 (92.82–99.84)	96.88 (81.52–99.54)	0.958
Negative	1 (nRPA)	31 (PCR/nPCR/RPA/nRPA)	32					
Total	90	32	122					

### Validation of RT-RPA in clinical samples

**Figure 3:**
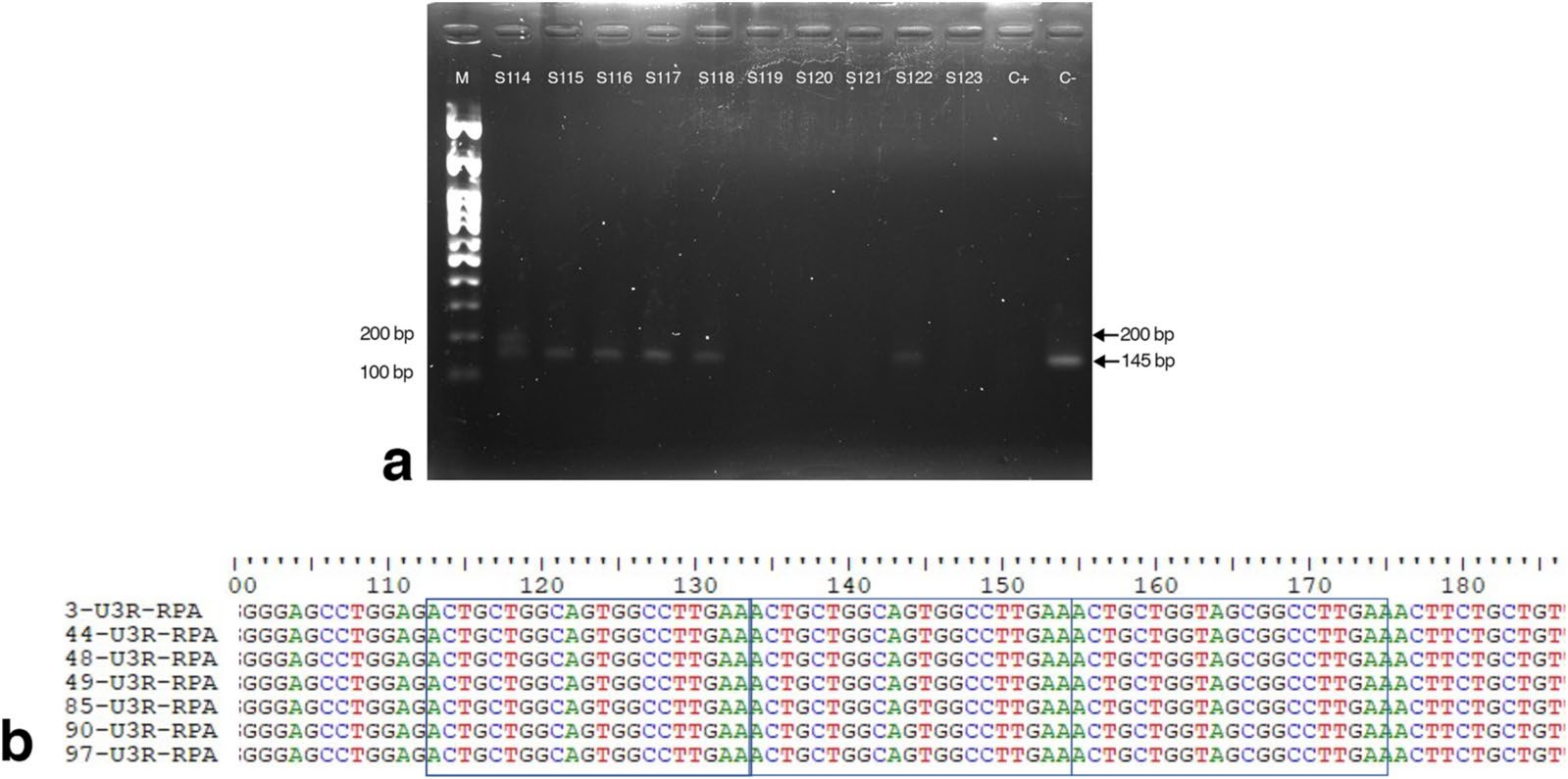
RT-RPA of clinical samples. (**a**) Representative image of agarose gel electrophoresis of RT-RPA products from clinical sample no. S114–S123. Legend: C+, positive DNA control; C−, no-template control; M, molecular weight marker. (**b**) Sequence analysis of the 200-bp RT-RPA amplicons from seven clinical samples.

The detection of FeLV RNA by RT-RPA was compared with its detection by rapid immunochromatographic assay or RT-PCR, and the results are shown in Table 4. The RT-RPA of 53 clinical samples gave the expected 145 bp product, while seven samples gave two amplicon bands, comprised of the expected 145 bp and an unexpected 200 bp product (Fig. 3). Sequence analysis of the amplicons from all seven samples revealed that the larger amplicon was due to a triplet repeat of a 21 bp sequence insertion between nucleotide positions 84–146 of the FeLV U3LTR (GenBank accession number AY374189.1), accounting for the extra size of the larger amplicon. Of 64 (52.46%) samples found to be FeLV positive by RT-PCR, there was one false negative results compared with the rapid immunochromatographic assay, and four compared with RT-RPA. The diagnostic performance of RT-RPA compared to the rapid immunochromatographic methods (Table 4) revealed a 95.24% (95% CI of 86.71–99.01) sensitivity, 100% (95% CI of 93.94–100.00) specificity, and kappa value of 0.951 (CI of 0.896–1.000). The diagnostic performance of the RT-RPA compared to RT-PCR revealed 93.75% (95% CI of 84.76–98.27) sensitivity, 100% (95% CI of 93.84–100.00) specificity, and a kappa value of 0.934 (CI of 0.871–0.997). The accuracies of RT-RPA vs. RT-PCR and vs. immunochromatography assay were 96.72% and 97.54%, respectively.

**Table 4:** Validation of clinical samples for FeLV testing by rapid immunochromatography, RT-PCR, and RT-RPA.

	Methods			Comparison	Sensitivity, % (95%CI)	Specificity, % (95%CI)	PPV, % (95%CI)	NPV, % (95%CI)	Kappa
	Rapid immune chromatography	RT-PCR	RT-RPA						
Positive (n)	63	64	60	RT-RPA vs. Rapid immune chromatography	95.24 (86.71–99.01)	100 (93.94–100)	100	95.18 (86.7–98.34)	0.951
Negative (n)	59	58	62	RT-RPA vs. RT-PCR	93.75 (84.76–98.27)	100 (93.84–100)	100	93.55 (84.88–97.4)	0.934
Total (n)	122	122	122

### Categorization of FeLV infective stages

The molecular diagnosis produced by all four methods was analyzed to determine the FeLV infective stage in symptomatic and healthy cats (Table 5). The progressive stage, in which FeLV RNA is found, was detected in 64 (52.46%) cats. The regressive stage, in which only FeLV provirus is found, was detected in 26 (21.31%) cats. Abortive or no infection was found in 32 (26.22%) cats. The highest incidence of FeLV infection was exhibited in lymphoma, leukemia, and other tumors at 87.5% (45/56), which were in the progressive [87.76% (43/49)] and regressive [12.24% (6/49)] stages of infection. For hematologic disorders, 8/10 cats had a FeLV infection, of which six displayed a progressive infection. Six of the nine FIV-infected cats were coinfected with FeLV, three with progressive and three with regressive infections. In addition, 4/8 cats with other infections, including Feline herpesvirus, Feline calicivirus, FIV, and *Mycoplasma haemofelis*, carried FeLV, two as progressive and two as regressive infections. Of cats with systemic diseases including kidney, heart, gastrointestinal, respiratory, and genital diseases, 56% (14/25) were infected with FeLV; 42.86% (6/14) cases were progressive and 57.14% (8/14) were regressive infections. Nine of 14 (64.29%) healthy cats that had visited the hospital for vaccination were found to have a FeLV infection. Both progressive and regressive stages were found in asymptomatic cats. In FeLV-related diseases, including lymphoma, leukemia, and hematologic disorders, FeLV infection was not detected in 12.5–20% of cats. In samples from cats with systemic diseases and other infections, 44–50% were negative for FeLV.

**Table 5:** Clinical characteristics and FeLV infective stages.

Clinical characteristics (n)	FeLV infected cats (%)	FeLV infective stages		
		Abortive/no infection (%^a^)	Progressive (%^b^)	Regressive (%^b^)
1. Lymphoma, leukemia, and other tumors (56)	49 (87.5%)	7 (12.5%)	43 (87.76%)	6 (12.24%)
2. Hematologic disorders (10)	8 (80%)	2 (20%)	6 (75%)	2 (25%)
3. FIV (9)	6 (66.67%)	3 (33.33%)	3 (50%)	3 (50%)
4. Other infections (8)	4 (50%)	4 (50%)	2 (50%)	2 (50%)
5. Systemic diseases: kidney, heart, gastrointestinal, respiratory, and genital diseases (25)	14 (56.00%)	11 (44%)	6 (42.86%)	8 (57.14%)
6. Healthy (14)	9 (64.29%)	5 (35.71%)	4 (44.44%)	5 (55.56%)
Total (122)	90 (73.77%)	32 (26.22%)	64 (52.46%)	22 (21.31%)

^a^% from each clinical characteristic.

^b^% from FeLV infection in each clinical characteristic.

## Discussion

In order for veterinarians to effectively manage the treatment of FeLV, and advice cat owners how best to take care of their pets, characterization of the different FeLV infective stages is useful. In this study, we developed novel nRPA and RT-RPA approaches to detect FeLV DNA provirus and RNA in circulating blood. The use of nRPA for FeLV provirus amplification increased the detection rate by about 45.8% (27/59) over that of p27 antigen detection. This increase would improve clinical diagnosis, because antigen detection has limited sensitivity for the detection of viremia in the progressive stage, resulting in a loss of latent detection. The detection of FeLV provirus using nucleic acid amplification based methods has been recommended as the gold standard due to its higher sensitivity and specificity^12^. The presence of endogenous FeLV was related to disease progression, viral recurrence, and vertical transmission in cats’ progeny.

FeLV-A is the most common subgroup in cats^24^. The nRPA and RT-RPA primers were designed to target U3LTR, which is the conserved fragment in all subgroups of FeLV^25^. nRPA targeting the U3LTR showed very high sensitivity and specificity, with almost perfect agreement with the results of nPCR and rapid immunochromatography detection methods. The concordance between nPCR targeting the *pol* gene and ELISA has previously been found to be substantial^26^, and nPCR has a 100-fold greater sensitivity than conventional PCR^27^. The sensitivity and specificity of RPA amplification could be enhanced, as in nPCR based methods. However, carry-over contamination must be prevented by physically separating the area of the primary PCR reaction from that of the secondary reaction. The primary RPA products were diluted to optimum concentration to produce high sensitivity and specificity. The dilution of the initial amplification products also reduced the carry-over problem. One negative control/16 nRPA reactions/nested nRPA run was checked for carry-over contamination. The positive control was used at the minimum concentration, and added as the last tube to each nested nRPA run to prevent contamination^28^.

The RT-RPA technique amplified FeLV RNA within 10 min. Longer incubation times yielded nonspecific bands only in the negative control. Bands with a smeared appearance were suggested to be primer dimers, which did not interfere with the interpretation of FeLV detection. Although unexpected 200 bp products were identified in seven samples, their sequences were similar to those of the expected amplicon, except for the presence of three 21 bp repeat sequences similar to those in FeLV subgroup A and the FeLV *v-myc* gene. The repeat elements naturally occur between the enhancer and promoter in the U3LTR during FeLV replication through error-prone reverse transcription, and by endogenous provirus recombination, including c-*myc* transduction. The LTR variants was associated to particular disease outcomes, especially thymic lymphomas, multicentric lymphomas, and other non-T cell diseases^29,30^.

The perfect kappa value indicated a significant correlation between the RT-RPA results with either the rapid immunochromatography or the RT-PCR assays. Similarly to insulated isothermal (ii)PCR and RT-iiPCR for point-of-need FeLV diagnosis, few discrepancies were revealed, especially in FeLV RNA detection^31^. Because RNA is fragile, some false negative results may result from poor quality or degenerated RNA, or a low viral load in the sample. The sensitivity and specificity of the three point of care FeLV p27 antigen test kits have previously been evaluated using qrtPCR as the gold standard, producing 63% sensitivity and 94% specificity for the SNAP Combo, 57% sensitivity and 98% specificity for Witness, and 57% sensitivity and 98% specificity for AntigenRapid^15^. The serological methods were unable to detect regressive disease in cats, and some kits produced false positive results due to interference by the antimouse IgG antibodies in cat sera^15^.

One of the advantages of the detection of FeLV provirus and RNA using nRPA and RT-RPA is the ability to achieve rapid amplification using general heating tools, with no need for thermal cyclers. However, the major drawback, the need for post amplification detection using gel electrophoresis, could be improved in the future by the use of SYBR green I or lateral flow strip (LFA) detection. A simple post RPA detection step in FeLV diagnosis is needed for FeLV diagnosis at a molecular level in veterinary clinics. Multiplexed RPA-LFA has been shown to be able to detect ESBL genes in *E. coli* isolated from pork meat and pig feces^32^. Recently, multidrug-resistant tuberculosis was rapidly detected by the naked eye, using allele-specific RPA combined with SYBR green I^33^. For RT-RPA, the TwistDx Basic RT kit is now not available for sale. However, a Basic TwistDX kit for DNA amplification can be used by adding an initial step involving a reverse transcriptase reaction such as RT-PCR to convert RNA to cDNA. The modified multiplexed RT-RPA by adding Moley murine leukemia virus reverse transcriptase and RNase inhibitor to the RPA pellet (Basic TwistDx kit, UK) successfully amplified and differentiated avian influenza viruses (AIVS) subtypes. Moreover, the combination of RT-RPA with capillary electrophoresis were simultaneously detected four AIVs genes with high sensitivity and high-throughput^34^. Recently, a one step RT-RPA by only adding ProtoScript II Reverse Transcriptase to the RPA pellet (Basic TwistDx kit, UK) could rapidly and sensitively detected SAR-CoV-2^35^. A combination of FeLV provirus and RNA testing using RPA assays is valuable for the diagnosis of the infective stages of FeLV before vaccination or blood transfusion. FeLV testing should be performed prior to blood donation to reduce the unintentional horizontal FeLV transmission. Nesina et.al.^10^ have demonstrated that naïve cats that received a blood transfusion from FeLV carrier cats developed FeLV-associated diseases, including nonregenerative anemia, T cell lymphoma, and secondary lymphoblastic leukemia.

We found that about 64% of asymptomatic cats visiting for vaccination become infected with FeLV. The existence of FeLV was revealed in 47.4% of healthy cats, using nested PCR^36^. In healthy cats not exposed to FeLV, 2% had regressive infections and 0.5% had progressive infections, while in a mixture of healthy and sick cats not exposed to FeLV 25% had regressive infections and 21% had progressive infections^37^. The administration of FeLV vaccine is ineffective in infected cats, and so makes cat owners become relatively incautious about preventing FeLV transmission. The rapid identification of the infective stage of FeLV would improve FeLV infection control by the timely isolation of an infected cats, maintaining separate food bowls and housing, and by promoting FeLV vaccination in high-risk cats, particularly in a multicat household.

Progressive FeLV infection has a poor prognosis, with a 80% fatality rate over 3 years^14^. Overall, FeLV infection was found in 87.5% of cats with lymphoma and leukemia, with 87.76% in progressive stages. The risk of lymphomas and leukemia was increased by up to 80% in cats infected with FeLV^18^. In a previous study, 15 out of 16 cats with myelodysplastic syndromes (MDS) were infected with FeLV. Nonregenerative anemia and thrombocytopenia were the most common hematologic abnormalities found in myelosuppression^13,38^. Coinfection of FeLV and FIV was found at about 7% in Madrid^39^ and at about 3.5% in Bangkok, Thailand during 2013–2014^4^, and at 2.8% in Mozambique, Southeast Africa in 2017^40^. Infection with FeLV induces immunosuppression, and so can lead to opportunistic infections. Other FeLV coinfections have been reported, including *Mycoplasma* spp., *Leishmania infantum*, Feline coronavirus, *Toxoplasma gondii*, *Dirofilaria immitis*, and *Bartonella henselae*^41^. FeLV causes immunopathological disorders and other systemic diseases, including glomerulonephritis, iridocyklitis or polyarthritis, enteritis, dermatosis, osteopetrosis, and exostosis^42,43^.

In conclusion, the RPA, nRPA, and RT-RPA methods developed in this study were validated against the gold standard of PCR, nPCR, and RT-PCR, producing perfect agreement. Both RT-RPA and RPA had similar sensitivity and specificity to PCR-based methods but with the additional advantages of rapid amplification and no need for an expensive thermal cycler. The characterization of the infective stages of FeLV using RPA-based assays could be set up onsite in veterinary hospitals to enhance the efficiency of FeLV diagnosis, which is important for prognosis and disease control. Rapid FeLV detection is important for the ultimate goal of disease prevention.

## Methods

### Ethical statement

The samples were obtained from The Small Animal Teaching Hospital, Faculty of Veterinary Science, Chulalongkorn University, Bangkok, Thailand, between January 2016 and December 2018. The study protocol was approved by Chulalongkorn University Animal Care and Use Committee (CU-ACUC 1831010). Written informed consent was obtained from each cat owner prior to the study. Blood was collected in EDTA tubes from 108 cats with clinicopathological abnormalities caused by FeLV infection, such as lymphoma, leukemia, hematologic disorders, FIV, and other infections, and from 14 healthy cats in a vaccination plan. All whole blood samples were tested for the FeLV p27 antigen using the Antigen Rapid FIV Ab/FeLV Ag test kit (BIONOTE, Korea) and kept at − 80 °C for further nucleic acid extraction.

All methods were carried out in accordance with relevant guidelines and regulations. The study was compliant with all relevant ethical regulations regarding animal research.

### Nucleic acid extraction

Two hundred microliters of whole blood was used for RNA and DNA extraction using QIAamp cador Pathogen (QIAGEN, Hilden, Germany) according to the manufacturer’s recommendation. Briefly, 20 µL of proteinase K and 100 µL of VXL buffer were combined and incubated at room temperature for 15 min. After adding 350 µL of ACB buffer, the solution was transferred to a QIAamp Mini column and centrifuged at 5000×*g* for 1 min. The column was washed twice with AW1 buffer and transferred to a new tube. The mixture of DNA and RNA was eluted by adding 70 µL AVE buffer, and centrifuged at 15,000×*g* for 1 min. The elution was aliquoted for DNA analysis and 30 µL was further purified for RNA using TURBO DNA-free kits (Ambion, Austin, TX, USA). All DNA and RNA samples were kept at − 80 °C for further analysis.

### Positive control of FeLV provirus and RNA

The U3 long terminal repeat (U3LTR) of the FeLV provirus was cloned into the pGEM-T Easy Vector (Promega, Madison, USA) and transformed into *Escherichia coli* JM109. The recombinant clones were extracted and utilized as a positive control for FeLV DNA provirus detection by PCR and nRPA assays. The Nobivac^®^ Feline 2-FeLV vaccine (Merck Animal Health/Intervet Inc, Nebraska, USA) was used for preparation of the FeLV RNA positive control for the RT-PCR and RT-RPA assays.

### Detection of FeLV DNA provirus by conventional nPCR

The U3LTR of exogenous FeLV DNA provirus was amplified using two sets of primers with the GoTaq Green kit (Promega Corporation, Madison, WI, USA). The first round of PCR primers were modified from Tandon et al.^44^ as follows; U3F1 RPA: 5′-AAACAGCAGAAGTTTCAAGGCCGCTACCAG-3′ and U3R1 RPA: 5′-CTGATGGCTCGTTTTATAGCAGAAAGCGCGCG-3′, to produce a 145-bp amplicon. The reaction was performed in 25 µL total volume comprised of 12.5 µL master mix, 0.2 µM of each primer, and 20 ng of template DNA. The thermal cycling conditions were 94 °C for 5 min followed by 40 cycles of 94 °C for 45 s, 65 °C for 1 min, and 73 °C for 1 min, followed by a final 73 °C for 5 min. The negative samples were then subjected to a second round of PCR reaction with two inner primers, as follows; U3F2 RPA: 5′-AAATTTCAAGGCCGCTACCAGCAGTCTCCAGG-3′ and U3R2 RPA: 5′-AGAAGCGAGAGGCGTGGGGATTGGTTAGTTAA-3′, to produce a 101-bp product. The second PCR reaction was comprised of the reagents as in the first round of PCR (except for the primers), using 1 µL of a tenfold dilution of the primary PCR product. The PCR conditions were 94 °C for 5 min followed by 40 cycles of 94 °C for 30 s, 65 °C for 45 s, and 73 °C for 45 s, followed by a final extension at 73 °C for 5 min.

### Detection of FeLV RNA using one step RT-PCR

The RT-PCR reaction was performed with the same primers used in the first round nPCR (above) using the AccessQuick™ RT-PCR System (Promega, Madison, USA) in a 25 µL reaction volume that consisted of 12.5 µL master mix, 0.3 µM of each primer, 1.5 µL of template RNA, and 0.5 µL of AMV reagent. Thermal cycling conditions were 45 °C for 45 min and 94 °C for 5 min, followed by 40 cycles of 94 °C for 45 s, 65 °C for 1 min, and 73 °C for 1 min, followed by a final extension at 73 °C for 5 min. The expected PCR product size was 145 bp.

### Detection of FeLV DNA provirus by nRPA

The nRPA was performed using a Basic TwistDX kit (TwistDX, Maidenhead, Berkshire, UK). The first and second sets of nested RPA primers were the same as those used in the nPCR above, and yielded the same sized products of 145 bp and 101 bp, respectively. The amounts of primary RPA products, primer concentrations, incubation temperature, and time were first optimized in preliminary trials. The first RPA reaction consisted of 0.24 µM of the U3F1 and U3R1 primers, 29.5 µL rehydration buffer, and 20 ng/µL template DNA in a total volume of 50 µL. The reaction mixture was added in lyophilized pellets and 2.5 µL of 280 mM magnesium acetate was added, mixed by inversion 8–10 times, and then incubated at 37 °C for 20 min. The 2 µL of primary RPA products from negative samples were diluted 100-fold and subjected to the second RPA reaction using the same nPCR primer set. The RPA conditions for the second round were unchanged, except that 0.18 µM of each primer was used, and incubation was carried out for 10 min only. The RPA products were purified using NucleoSpin^®^ Gel and PCR Clean-up kits (MACHEREY-NAGEL, Düren, Germany) as per the manufacturer’s recommendation, before analysis using 1.5–2% (w/v) agarose gel electrophoresis with visualization by a ChemiDoc XRS gel photo documentation system (Bio-Rad, Hercules, CA, USA).

### Detection of FeLV RNA using RT-RPA

TwistDx Basic RT kits were used with 0.12 µM of U3F1 and U3R1 primers. The primer concentrations, temperature, and incubation time were first optimized in preliminary trials. The reaction mixture was prepared in a final volume of 50 µL composed of rehydration buffer, 10 ng/µL RNA template, and 100 unit of RNAse inhibitor. Lyophilized pellets were solubilized in the reaction mixture, and 2.5 µL of 280 mM magnesium acetate was added and mixed by inversion 8–10 times. The optimum conditions were 40 °C for 10 min. The RT-RPA products were purified using NucleoSpin^®^ Gel and PCR Clean-up (MACHEREY-NAGEL) and analyzed for the presence of the 145 bp product using 1.5% (w/v) agarose gel electrophoresis.

### Clinical sample testing

All 122 clinical samples from symptomatic and vaccinated cats were tested using nRPA and RT-RPA, respectively. The TwistDx basic kit and TwistDx Basic RT kit were employed, using the conditions described above.

### Data analysis

The results of the nRPA were compared with those from the PCR and nPCR assays, while the results from the RT-RPA were compared to those from the RT-PCR and immunochromatography assays. Inconsistent results between RPA, nRPA, PCR, nPCR and RT-RPA, RT-PCR, and immunochromatography assay were repeated using all methods to confirm the results. The sensitivity and specificity were determined using the MEDCALC^®^ software (https://www.medcalc.org/calc/diagnostic_test.php). To assess the concordance level, the kappa value was calculated with 95% CIs using QuickCals software (https://www.graphpad.com/quickcalcs/kappa1)^45^.

## Supplementary Information

Supplementary Figure S1.

## Data Availability

All data generated or analysed during this study are included in this published article. The full-length gel in Fig. 1a was presented in supplemental file Fig. 1.
