# Supplementary material for: Rapid characterization of feline leukemia virus infective stages by a novel nested recombinase polymerase amplification (RPA) and reverse transcriptase-RPA
Source: Sci Rep. 2021 Nov 11;11:22023. doi: 10.1038/s41598-021-01585-9 (PMC8586258; doi:10.1038/s41598-021-01585-9)
Supplement: Supplementary file 1 — Supplementary Figure S1. [file 41598_2021_1585_MOESM1_ESM.docx]

a
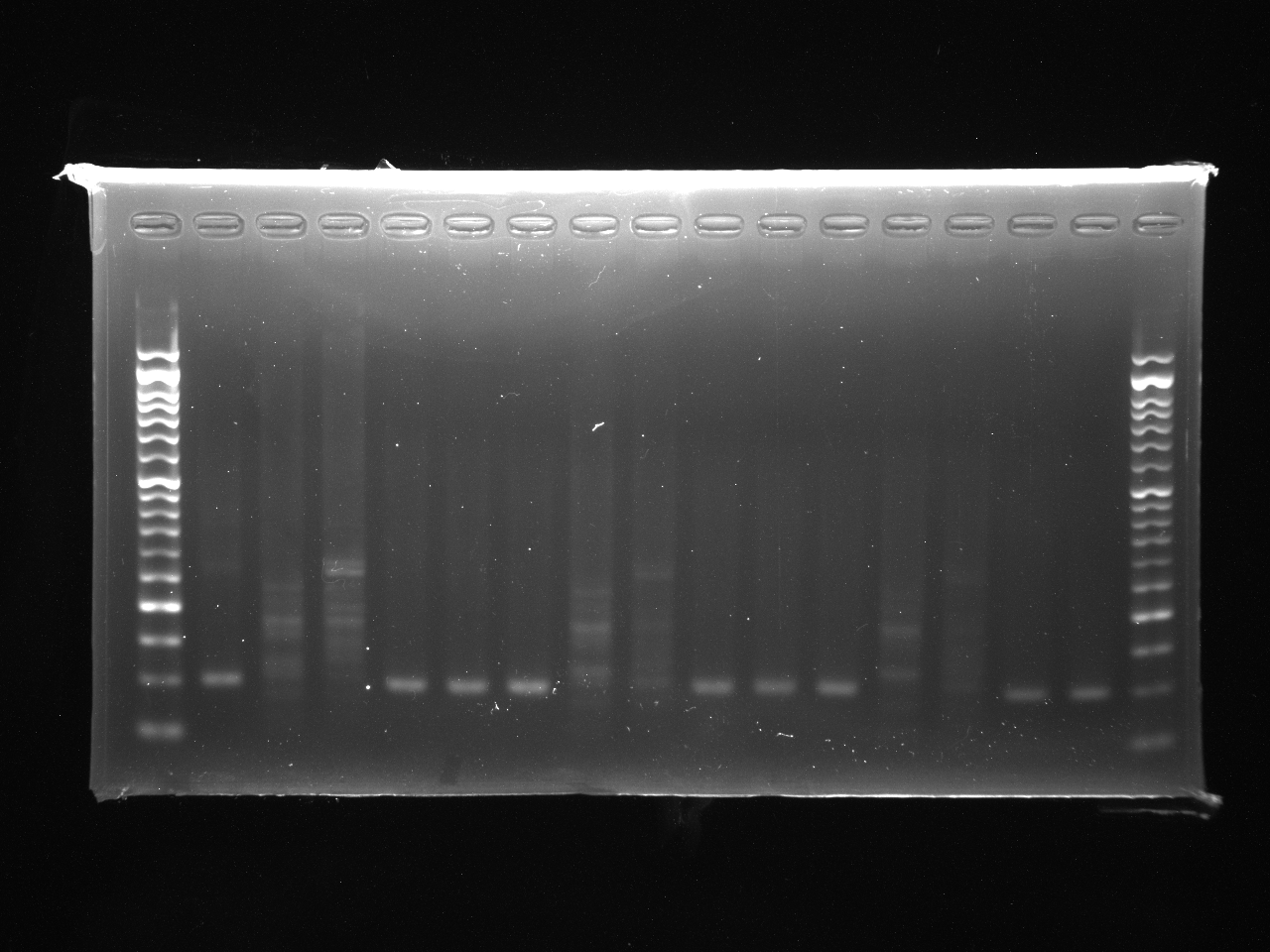


M 10 μM 7.5 μM 5 μM M

C+ C- 1 7 99 C+ C- 1 7 99 C+ C- 1 7 99

50 bp

150 bp

100 bp

Supplementary Figure 1. Optimization of FeLV DNA provirus detection by nRPA. Representative gel images showing the results from the nRPA reaction at different (**a**) inner primer concentrations (10, 7.5, and 5 µM) incubated at 37 ^o^C for 20 min. Legends: C+, positive DNA control; C-, no template control; 1, 7, 9, clinical samples; M, molecular weight marker.
